# Positive Human Contact and Housing Systems Impact the Responses of Piglets to Various Stressors

**DOI:** 10.3390/ani11061619

**Published:** 2021-05-30

**Authors:** Megan E. Hayes, Lauren M. Hemsworth, Rebecca S. Morrison, Alan J. Tilbrook, Paul H. Hemsworth

**Affiliations:** 1 The Animal Welfare Science Centre, Faculty of Veterinary and Agricultural Sciences, The University of Melbourne, Parkville, VIC 3010, Australia; lauren.hemsworth@unimelb.edu.au (L.M.H.); phh@unimelb.edu.au (P.H.H.); 2 Rivalea Australia Pty Ltd., Corowa, NSW 2464, Australia; RMorrison@rivalea.com.au; 3 Centre for Animal Science, Queensland Alliance for Agriculture and Food Innovation, The University of Queensland, St Lucia, QLD 4072, Australia; a.tilbrook@uq.edu.au

**Keywords:** pig welfare, early life experiences, housing, handling, positive human contact

## Abstract

**Simple Summary:**

Early life experiences such as contact with humans, maternal care and the physical environment can play a substantial role in shaping behavioural and physiological responses to stress. This experiment studied the effects of lactation housing systems and human interaction on stress in young pigs. We hypothesised that piglets handled in a positive manner and reared in loose farrowing and lactation pens with increased opportunity for interaction with their dam, greater space and more complexity in their physical environment have improved stress resilience than piglets reared in traditional farrowing crates with routine contact from stockpeople. In both housing systems, providing regular opportunities for positive human interaction reduced piglets’ fear of humans and routine husbandry procedures imposed by humans, and reduced the number of injuries obtained after weaning. However, contrary to the expected findings, piglets from loose farrowing and lactation pens were more reactive to capture by a stockperson, more fearful of novel and human stimuli, had more injuries during the lactation period and were more likely to perform behaviours which may be indicative of reduced coping after weaning. Whether these effects are specific to the loose farrowing and lactation system studied in this experiment or are reflective of other loose systems requires further research.

**Abstract:**

This experiment studied the effects of lactation housing systems and human interaction on piglets’ responses to routine stressors. Forty litters of piglets were reared in either a standard farrowing crate (FC) or a loose farrowing and lactation pen (LP; PigSAFE pen) and received either routine contact with humans (C) or regular opportunities for positive human contact (+HC; 3 min of patting, stroking and scratching 5 times/week). Behavioural and physiological responses to routine husbandry procedures, weaning, novelty and humans were studied in addition to effects on piglet growth, injuries and survival. Compared to C piglets, +HC piglets vocalised for shorter durations (*p* = 0.018) during husbandry procedures and showed a lower intensity of escape behaviour during iron injection (*p* = 0.042) and oral vaccination (*p* = 0.026) at 3 d of age, capture at 2 wk of age (*p* < 0.001), and intramuscular vaccination (*p* = 0.005) at 3 wk of age. +HC piglets at 2 wk of age were faster than C piglets to approach (*p* = 0.048) and interact (*p* = 0.042) with a stationary unfamiliar human. Compared to LP piglets, FC piglets showed a lower intensity of escape behaviour during capture and iron administration by a stockperson at 3 d of age (*p* = 0.043). FC piglets at 2 wk of age were faster than LP piglets to approach (*p* = 0.005) and interact (*p* = 0.027) with a novel object and approach (*p* = 0.009) and interact (*p* = 0.008) with an unfamiliar human. FC piglets had fewer injuries than LP piglets at 2 wk of age (*p* = 0.004). +HC pigs had fewer injuries than C pigs after weaning (*p* = 0.003). After weaning there were more pigs from LP than FC observed to be upright (both stationary, *p* = 0.002 and walking, *p* = 0.024), vocalizing (*p* = 0.004), nosing another pig (*p* = 0.035) and nosing the pen floor (*p* = 0.038). There were no significant effects on neutrophil:lymphocyte ratios or plasma cortisol concentrations 1.5 h after weaning. However, 25 h after weaning +HC pigs had higher haptoglobin concentrations than C pigs (*p* = 0.002), and C/LP pigs had higher cortisol concentrations than +HC/LP and C/FC pigs (*p* = 0.012). There were no significant effects on piglet growth, the number of piglets born alive or the number stillborn, however there were more piglets weaned from FC than LP (*p* = 0.035). The results from this experiment raise questions that require further research on the ability of pigs reared in loose pens to cope with stressors such as exposure to humans, novelty, husbandry procedures and weaning. This experiment also provides evidence that regular positive human interaction reduces pigs’ fear of humans and husbandry procedures imposed by stockpeople. More research is required to determine if any of these effects are sustained long-term.

## 1. Introduction

Commercial pigs are exposed to several stressful situations as part of routine production, including painful husbandry procedures, close contact with stockpeople, exposure to novel environments, weaning and mixing with unfamiliar pigs. Stress resilience, which refers to the ability of an individual to cope with and recover from stress [1], has obvious implications for the pig industry as an impaired ability of pigs to cope with these routine stressors is likely to negatively affect pig welfare and productivity based on studies in non-human primates and rodents [2,3,4]. Stress resilience can be shaped by several early-life environmental inputs, and for pigs, the physical environment, maternal care from the sow and interactions with humans are likely candidates.

Farrowing crates remain the most widely used system for rearing pigs during the lactation phase, although increasing concern for animal welfare has led to the design of several alternative farrowing and lactation housing systems, which allow free movement of the sow. One example is the PigSAFE pen (Piglet and Sow Alternative Farrowing Environment), a loose farrowing and lactation system that was designed to optimise sow welfare and piglet survival while maintaining ease of management and commercial viability [5]. PigSAFE pens offer greater space and contain features such as sloped walls, varied flooring and separate sections for feeding, nursing and elimination, and as such are considered to be a more complex environment than the traditional farrowing crate [6]. In addition, PigSAFE pens offer greater opportunity for sow–piglet interaction, which may have implications for the level of maternal care piglets receive. Sows in other loose lactation systems show improved maternal behaviour, as evident by increased responsiveness to piglet vocalisations and more frequent interactions with their piglets in comparison to sows from farrowing crates [7,8,9]. In loose housing systems such as PigSAFE, the increased environmental complexity and the greater opportunity for maternal care may contribute to improved stress resilience in piglets.

While research on the PigSAFE system is sparse, there is evidence that pigs reared under different maternal and/or physical conditions vary in their responses to stressors routinely encountered in production. Pigs from farrowing crates showed greater cortisol responses after ear tagging [10] and restraint [11] compared to pigs from outdoor pens, and greater cortisol responses after transport compared to pigs from larger crates and pens enriched with straw [12]. The ratio of neutrophil to lymphocyte cells after husbandry procedures was higher in pigs from barren environments than pigs from environments enriched with newspaper, soil, balls and rope [13]. Play behaviour was performed more frequently [6,9] and occurred earlier in life [6] in piglets in loose pens, which suggests the farrowing crate environment negatively impacts the development of normal social skills. This can affect how pigs respond to social stressors, since after mixing with unfamiliar pigs at weaning, belly nosing and manipulative behaviours were performed more frequently by pigs from farrowing crates than loose pens [14]. Furthermore, pigs reared in loose pens spent more time exploring food after weaning [14], and tended to show improved growth compared to piglets from farrowing crates [15]. Acute phase proteins such as haptoglobin have also been used to study stress in pigs [16,17] and may provide information on the magnitude of the stress response to weaning [18].

Many challenges faced by commercial pigs involve close contact or handling by stockpeople, and thus responses to these challenges are affected by the level of fear pigs have towards humans. Piglets that had experience with a handler who moved unpredictably and shouted during routine feeding and inspections demonstrated less resting, and more escape attempts and agonistic interactions after weaning, in comparison to piglets that had experience with a handler who moved carefully and used a soft tone of voice [19]. Talking softly and imposing gentle tactile contact such as stroking and scratching upon approach is perceived positively by pigs [20] and reduces fear of humans [20,21,22]. Patting and stroking during suckling bouts reduced the duration of escape behaviour in piglets during husbandry procedures conducted at 2 d of age and capture at 15 d of age [23], and daily patting and scratching reduced the avoidance responses of sows to stockpeople imposing pregnancy testing and vaccination [24]. Pigs can remember positive interactions with humans for at least 5 wk [25], and when these interactions take place early in life the effects may be sustained for up to 18 wk [26].

The effects of providing pigs with opportunities for positive human interaction extend beyond reducing fear of humans. Piglets that were stroked while being held showed reduced fear of tactile contact by familiar and unfamiliar people, but also performed more play behaviour and vocalised less in a novel arena, suggesting that the handling treatment reduced both fear of humans and novel situations [27,28]. Positive handling has also been reported to reduce tail biting behaviour in weaner pigs, although the handling treatment in this experiment involved attracting piglets with chopped straw which is a potentially confounding factor [29]. Weight gain was higher in piglets habituated to human handling [30] and human presence [27]. The presence of a familiar human after social isolation reduced the duration and frequency of piglets’ vocalisations, which suggests that familiar people may even buffer stress when pigs are exposed to challenging situations [31].

Since maternal care, the physical environment and interactions with humans can impact stress resilience, the aim of this experiment was to study the effects of farrowing and lactation housing systems and positive human contact on piglets’ responses to routine stressors. The hypothesis for this experiment was that piglets reared in loose farrowing and lactation pens (PigSAFE pens) with opportunities for positive human interaction show improved stress resilience compared to piglets reared in traditional farrowing crates with routine contact from stockpeople.

## 2. Materials and Methods

This experiment was conducted at a large commercial piggery in NSW, Australia. All animal procedures were conducted with prior institutional ethical approval under the requirements of the New South Wales Prevention of Cruelty to Animals Act 1985 in accordance with the National Health and Medical Research Council/Commonwealth Scientific and Industrial Research Organization/Australian Animal Commission Code of Practice for the Care and Use of Animals for Scientific Purposes.

### 2.1. Animals and Treatments

Forty litters of piglets from primiparous sows (Landrace × Large White) were studied from birth until 2 d after weaning in a 2 × 2 factorial design, with the main effects as listed below and detailed in subsequent sections:

1. Housing system
  - FC—Farrowing crate
  - LP—Loose pen
2. Human contact treatment
  - +HC—Positive human contact
  - C—Routine human contact

#### 2.1.1. Housing Systems during the Farrowing and Lactation Period

**Figure 1:**
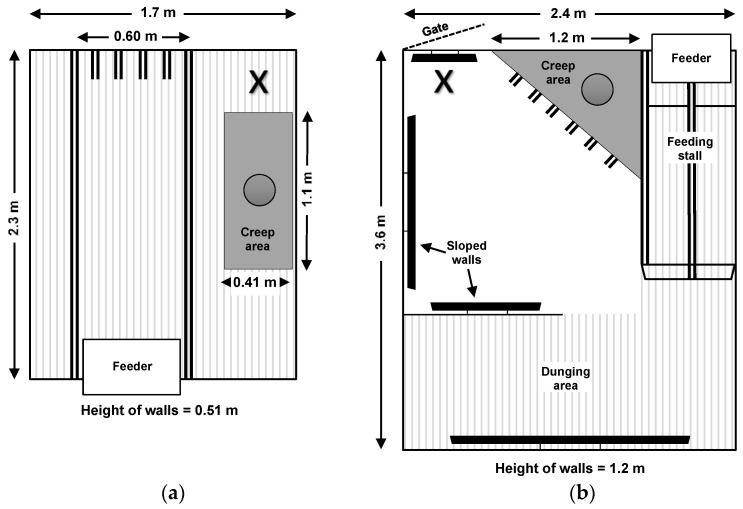
Layout and dimensions of the two housing systems: (**a**) farrowing crate (FC); (**b**) loose pen (LP). X denotes the position of the experimenter during the imposition of the positive human contact treatment.

The layouts of both housing systems studied are depicted in Figure 1. The two housing systems were in one shed but in separate rooms adjacent to one another. The footprint of each farrowing crate was 2.3 × 1.7 m, with an internal space for the sow of 2.3 × 0.60 m, which allowed the sow to stand or lay down, but not turn around. Farrowing crates had a creep area that was heated by an overhead lamp and contained a 1.1 × 0.41 m solid floored mat. The rest of the floor was slatted steel (10 mm width between slats). The walls of each farrowing crate were solid and 0.51 m high, allowing sows and piglets to have visual contact with stockpeople in the aisles.

The design of the loose pen system studied was PigSAFE (Piglet and Sow Alternative Farrowing Environment). Loose pens were 3.6 × 2.4 m and allowed free movement of the sow during farrowing and the entire lactation period. Loose pens contained a triangular creep area only accessible to piglets, which had an overhead lamp and a removable solid roof. The sows’ feeder was located within a stalled area where the sow could be confined temporarily if stockpeople required access to the pen or piglets, although stockpeople very rarely used the feeding stall for this purpose. The flooring in the central area, including the creep, was solid plastic while the flooring in the back-dunging area and the feeder stall was slatted plastic (MIK Rubin flooring, 10 mm width between slats). The walls of the loose pens were solid and 1.2 m high, which restricted piglets’ visual contact with humans in the room unless they were standing directly in front of the pen. The loose pens contained barred windows which allowed interaction between adjacent sows. Interaction between adjacent piglets was restricted early in life but was possible at 2–3 wk of age once piglets were large enough reach the windows. The walls of each pen were also sloped to reduce the incidence of piglets being crushed by the sow.

#### 2.1.2. Human Contact Treatment during the Lactation Period

Piglets from the C treatment received only routine contact with stockpeople associated with regular husbandry and management. The +HC treatment also involved routine husbandry and management as in the C treatment, in addition to the opportunity to interact with a female experimenter five days per week from 1 d of age until weaning. The +HC treatment involved the experimenter gently patting, stroking and scratching piglets that approached her and piglets sleeping in the creep area for a duration of 3 min. Piglets were patted, stroked and scratched on the back, the abdomen and behind the ears. The +HC treatment was imposed at the litter level, in the morning after sows had been fed (approximately between 7:00 and 9:00 h) mainly by one female experimenter. However, on six days an additional female experimenter assisted in imposing the treatment on half of the +HC litters, as other experimental activities had to be completed so the total time imposing treatment needed to be reduced.

To impose the +HC treatment in farrowing crates, the experimenter slowly entered and crouched down at the back of the crate in front of the creep area (see location marked as “X” in Figure 1a). To impose the +HC treatment in loose pens, the experimenter stood outside the pen and secured the sow in the feeding stall before slowly entering the pen and crouching next to the creep area (see location marked as “X” in Figure 1b). To minimise distress to sows from being secured in the stalls, and consequently, to minimise disruption to the piglets during +HC imposition, all sows from loose pens were trained to voluntarily enter the feeding stalls. This training began 1 d after sows had been introduced to the pens and involved offering food rewards (5–10 small chocolates in the feeder) after the sow entered the stall, and securing the sow for increasing durations, starting with 30 s on the first day of training and building up to 10 min on the last day. In the event a sow from a +HC pen did not voluntarily enter the stall prior to treatment imposition, the experimenter quietly lifted the creep roof, squatted outside the pen and extended their hand inside the creep area to impose the positive handling treatment. The handling treatment was delivered this way in 20% of instances. Whenever any sow from a loose pen was offered food rewards, either during the training period or occasionally prior to +HC treatment delivery to lure the sow into the feeding stall, all other sows including those from farrowing crates were provided with the same quantity. During imposition of the positive handling treatment sows from C pens were always secured in the feeding stalls for the same duration as those from +HC pens, which was never longer than 10 min.

### 2.2. Human Contact Treatment Allocation

**Figure 2:**
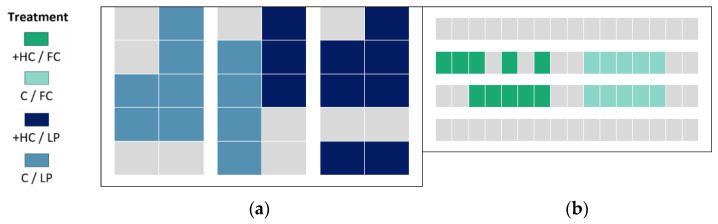
Allocation of positive human contact (+HC) and routine human contact (C) litters: (**a**) loose pen (LP) room; (**b**) farrowing crate (FC) room. Grey boxes in both housing systems represent non-experimental litters.

C and +HC litters were allocated to opposite ends of each room in order to minimise any carry-over effects of the positive handling treatment. In particular, it was important to minimise the amount of visual exposure to humans that C litters received through the imposition of the +HC treatment, as fear of humans is reduced in pigs that observe positive handling of other pigs [32]. Thus, in the farrowing crate room where 2 parallel rows of crates were used, non-experimental litters (also from first parity sows) were allocated in between +HC and C litters (Figure 2b). In the loose pen room, which contained 30 pens in three blocks of back-to-back pens, 20 experimental litters were positioned in the three blocks as shown in Figure 2a with non-experimental litters in the other pens. In this room there was a reduced chance of carry-over effects between +HC and C litters as the loose pens had high walls that restricted piglets’ visual contact outside of their own pen.

### 2.3. Management of Animals

At least 5 d prior to expected parturition, sows were moved from group gestation pens into one of the two farrowing and lactation housing systems. As per standard practice at the piggery, sows in farrowing crates had a cotton rope suspended in front of the crate from 1 d after entry to the farrowing house until 1 d after parturition, while sows in loose pens were provided with a small amount (approximately two large handfuls) of sawdust in the central area of the pen 2 d prior to expected parturition. No other bedding or enrichment was provided to sows or piglets after farrowing. Sows and piglets from both housing systems had ad libitum access to water. While farrowing crates and loose pens were in separate but adjacent rooms, the animals were managed by the same group of male and female stockpeople. All animals received twice daily health and welfare checks by stockpeople. This involved a stockperson visually inspecting the animals and entering the pen when necessary, for example to assess sow teat function or remove a dead piglet. Sows were hand-fed by stockpeople twice per day, and piglets were hand-fed creep food once per day after 14 d of age. The farrowing spread was 7 days across all treatments and litters were not equalised for size or balanced for equal sex ratios. Cross fostering occurred on a minimal basis, always within the same housing system and within the first 24 h of life. The total number of piglets fostered was 6 in the FC treatment and 7 in the LP treatment. All piglets underwent routine husbandry and management as per standard commercial practice, including processing (intramuscular iron injection, administration of an oral coccidiosis treatment and tail clipping with a gas cautery iron) at 3 d of age, administration of an intramuscular vaccination (porcine circovirus-associated disease) at 3 wk of age and weaning at 22 d of age (SD = 2.4). Weaning age did not differ between treatment groups. At weaning all pigs from the same sex, housing system and human contact treatment were moved to the weaner facility together and then separated into groups of 10 pigs in 3.0 × 1.5 m pens with ^3^/_4_ slatted steel flooring (10 mm width between slats). Pens were spread across two adjacent rooms with pigs from the same sex and treatment group generally housed in nearby pens. After weaning, *n*_+HC/FC_ = 11, *n*_C/FC_ = 8, *n*_+HC/LP_ = 7 and *n* _C/LP_ = 9 single sex pens. The pigs were not individually identifiable, however, prior to weaning all were marked with stock spray which enabled litter identification once pigs had been mixed post-weaning.

### 2.4. Measurements

#### 2.4.1. Behavioural and Physiological Responses to Routine Husbandry Procedures

Processing occurred at 3 d of age and was carried out by two stockpeople. One stockperson entered the farrowing crate or loose pen and lifted a piglet, injected an iron supplement intramuscularly and passed the piglet to a second stockperson who administered an oral coccidiosis treatment and placed the piglet into a trolley. In loose pens, a solid stockboard was held to visually separate and protect the stockperson from the sow. Once all piglets from the litter were in the trolley, the first stockperson picked each piglet up and clipped the tail with gas heated cautery clippers before returning the piglet to the home pen. Direct observations were used to record the behavioural responses of all piglets to each of these procedures. The intensity of piglet escape behaviour was scored using the following ordinal scale adapted from Leidig et al. [33]: 0—no movement; 1—movement of one or two limbs; 2—movement of multiple limbs and the spine; and 3—movement of multiple limbs and the spine but with high intensity, repeatedly. The same two observers scored each litter, with the first observer responsible for scoring the intensity of escape behaviour in response to capture by the stockperson and iron injection (these events almost occurred simultaneously, so only one score was given), and tail clipping. The second observer was responsible for scoring escape behaviour during administration of the oral treatment. A third observer recorded piglets’ vocalisations throughout processing by holding a microphone (Samson Meteor USB microphone) between the two stockpeople conducting processing, so that it was always less than 1 m away from piglets. The microphone was connected to a laptop running Raven Pro sound analysis software [34], which was used to record the vocalisations and later determine the total number and duration of vocalisations for each litter. Blood samples for subsequent analysis of cortisol were collected 45 min after processing from 2 males and 2 females selected from each litter (see subsequent section “Sample Collection Details and Assay Characteristics” for further detail). Four piglets from each litter were selected for sampling on the basis of the first piglet alternately sighted in the front and back of the pens.

At 3 wk of age, all piglets received an intramuscular vaccination (porcine circovirus-associated disease) and their behavioural responses to capture and vaccination were scored by one observer using the previously mentioned scale. This observer was one of the people responsible for scoring the behavioural responses of piglets at processing.

#### 2.4.2. Behavioural Responses to Novelty and Humans in a Standard Test

At 2 wk of age, the behavioural responses to novel and human stimuli were assessed in 2 male and 2 female piglets selected from each litter. Four piglets from each litter were selected for sampling on the basis of the first piglet alternately sighted in the front and back of the pens. The four piglets from a litter underwent testing together, which involved consecutive 1 min exposures to the following conditions: empty novel arena located adjacent to the home pen; novel object introduced to arena; human hand introduced to arena; unfamiliar human standing inside arena. The 1.8 m × 0.60 m × 0.60 m novel arena was constructed of black wooden board and was portable, allowing testing to take place 1 m away from each FC and LP. Painted lines were used to mark thirds on the floor and a Go-Pro camera was mounted above the arena. Piglets were individually lifted from the home pen and gently placed at one end of the arena by an experienced technician. Once all 4 piglets had been placed inside, they were left in the empty arena for 1 min. A female experimenter, unfamiliar to the piglets, then slowly approached and presented an orange traffic cone at the opposite end of the arena to where piglets had been initially placed. After 1 min, the experimenter approached, removed the traffic cone and squatted side-on outside the arena while extending their hand inside. After a further minute the experimenter stood up and stepped inside the arena, remaining stationary for the final minute of the test. Each piglet was then individually returned to the home pen. Direct observations by one observer were used to record the intensity of escape behaviour of piglets during capture from the home pen prior to testing and capture from the arena at the conclusion of the test, using the scale previously described (see previous section “Responses to Routine Husbandry Procedures”). Using video footage the number of entries each piglet made into different sections of the empty arena were recorded. Additionally, the following behavioural responses to the novel object, human hand and the standing human were recorded: latency to approach within 0.6 m, time spent within 0.6 m, latency to initiate tactile interaction (sniffing, nosing, chewing or stepping on stimulus), and the number of tactile interactions with the stimulus. A maximum response time of 1 min was given if a piglet did not approach or interact with the stimulus.

#### 2.4.3. Behavioural and Physiological Responses to Weaning

At weaning, pigs were mixed into group pens of 10 animals of the same sex from the same housing system and human contact treatment. Direct observations were used to record the behaviour of pigs for 30 s at 15 min, 1 h, 2 h, 3 h, 4 h, 24 h, 25 h and 26 h after weaning. One observer stood 2 m away from each pen and recorded the number of pigs upright (either stationary or walking), nosing the pen floor (upwards and downwards movement of snout against the floor), nosing a pen mate (upwards and downwards movement of the snout against another pig’s head or body) and vocalising, as well as the number engaging in aggression (knocking, biting or pushing a pen mate), tail biting (mouthing or chewing the tail area of another pig) and play (energetic running or hopping, pivoting on the spot or tossing the head) at each time point. Blood samples were obtained from 4 pigs selected from each pen 1.5 h after weaning for subsequent analysis of plasma cortisol and total neutrophil and lymphocyte cell counts, and 25 h after weaning for subsequent analysis of plasma cortisol and haptoglobin (see subsequent Section “Sample Collection and Assay Characteristics” for further detail). Four pigs from each pen were selected for sampling on the basis of the first pig alternately sighted in the front and back of the pens.

#### 2.4.4. Sow Reproductive Performance and Piglet Growth, Injuries and Survival

The litters were weighed at 3 d and 18 d of age. To assess piglet injuries, scratches and abrasions were scored at 2 wk of age on the 2 males and 2 females from each litter selected for behaviour testing and 2 d after weaning on 4 pigs selected from each pen. Each selected pig received one score for scratches and abrasions on the body and head using the following scale as first described by Widowski et al. [35]: 0—no scratches or skin loss were evident; 1—one to three small (2 cm) scratches or areas of abraded skin were evident; 2—one to three larger (>2 cm) scratches or areas of abraded skin were observed; and 3—more than three scratches (usually >2 cm) or larger areas of superficial skin loss. Sow reproductive performance and piglet survival were assessed through records of the total number of piglets born alive, stillborn and weaned.

#### 2.4.5. Sample Collection Details and Assay Characteristics

Blood sampling was carried out by two teams of experienced technicians who collected samples from pigs in adjacent pens simultaneously. In each team, there was one person to pick up and hold the piglet inverted, one person to collect the sample and another to record the time and order of sampling within the pen. Piglets were not individually identifiable and were selected for sampling on the basis of the first pig alternately sighted in the front and back of the pens, although obvious runts were excluded. All samples were obtained within 2 min of picking up the piglet. Blood was collected via jugular venipuncture into 4 mL EDTA-coated tubes (BD Vacutainer, NSW, Australia). Tubes were inverted 8–10 times and placed immediately on ice after collection. Samples obtained after processing were collected between 11:00 and 14:00 h, and samples obtained after weaning were collected between 9:00 and 11:00 h. Samples for analysis of cortisol and haptoglobin were centrifuged for 10 min at 2000× *g* and plasma was transferred to polypropylene tubes and frozen at −20 °C until later analysis. Cortisol was quantified using a commercially available ELISA kit from Enzo Life Sciences (ADI-900-071), with a normal detectable range of 156–10,000 pg/mL. Samples were assayed at 1:16 dilution. Haptoglobin was quantified using a commercially available ELISA kit from Abcam (ab205091) with a normal detectable range of 6.25–400 ng/mL. Samples were assayed at 1:40,000 dilution. The intra- and inter-assay co-efficients of variation were less than 10% for the cortisol and haptoglobin assays. Samples collected for haematology were transported to a commercial laboratory where the absolute numbers of neutrophil and lymphocyte cells were counted using a Sysmex XT-2000i analyser (Sysmex, Kobe, Japan). The ratio of neutrophil to lymphocyte cells was then determined.

### 2.5. Statistical Analysis

All analyses were conducted in GenStat for Windows 18th Edition [36]. Unless otherwise stated, data were analysed using linear mixed models with the REML (Restricted Maximum Likelihood) directive, with human contact treatment, housing system and the interaction between the two as fixed effects. Sex was also included as a fixed effect for analysis of injury scores, behaviours observed after weaning, and physiological measurements collected after processing and weaning. For behavioural responses in the behaviour test, the sex of the piglets was not identifiable from video footage, however, 2 males and 2 females were tested from each litter and thus sex was balanced across treatments. For behavioural responses to husbandry procedures, the sex of the piglets was not known during the observation period. Litter and age were included as random effects in the model if they returned a variance component greater than 0. Age was excluded from the model for measurements obtained when all litters were of equal age, including responses to husbandry procedures and litter weights. For measures obtained post-weaning, post-weaning pen was included as an additional random effect. During processing piglet vocalisations were recorded continuously for each litter, and so the total number and duration of vocalisations was divided by the litter size to estimate the number and duration of vocalisations per piglet. Similarly, weights were obtained at 3 d and 18 d of age at the litter-level. The total weight of the litter was divided by the litter size on the day of weighing to estimate the weight of each piglet.

All data were checked for outliers, normality and homoscedasticity via visual inspection of residual plots. Logarithmic (base 10) transformations were applied to the number of vocalisations recorded during processing and to all physiological measurements collected after processing and weaning to correct the skewed distribution of the residuals. Using the GLMM procedure, generalised linear mixed models using a Poisson distribution with a logarithmic link function were used to analyse the number of tactile interactions piglets made with stimuli in the standard test. Generalised linear mixed models using a binomial distribution with a logit link function were used to analyse the number of pigs engaging in key behaviours after weaning. As behaviours after weaning were observed at several time points, time of the observation was added as an additional fixed effect. The behaviour of nosing the pen floor was only observed once on the first day of observations, and thus only data from observations at 24 h, 25 h and 26 h after weaning were included in the analysis of this behaviour. For all behaviours observed after weaning, odds ratios were calculated by exponentiating the difference between treatment group means. The odds ratio represents the odds of a pig from one treatment group partaking in the behaviour over a pig from another treatment group, thus when the odds ratio is 1 the chance of pigs from different treatments partaking in the behaviour measured is equal. Where transformations were applied or where generalised linear mixed models were fitted, back transformed means are presented.

The effects of human contact treatment on the number of piglets born alive and the number of piglets stillborn were not tested as the positive handling treatment only began at 1 d of age (the day after farrowing), and thus only effects of housing system were tested on these measurements. Even after transformations were applied, the residuals were markedly skewed for the number of piglets stillborn and so a non-parametric Mann–Whitney U (Wilcoxon rank-sum) test was used to test for effects of housing system on the number of piglets stillborn.

## 3. Results

### 3.1. Responses to Routine Husbandry Procedures at 3 d and 3 wk of Age

#### 3.1.1. Behavioural Responses to Processing and Vaccination

During processing at 3 d of age, +HC piglets showed a lower intensity of escape behaviour to capture and iron injection compared to C piglets (mean score; 1.32 vs. 1.70; *F*_1,36_ = 4.43; *p* = 0.042) and to administration of an oral treatment (1.35 vs. 1.68; *F*_1,36_ = 5.41; *p* = 0.026; Table 1). The intensity of escape behaviour to capture and iron injection at processing was also lower in FC piglets compared to LP piglets (1.32 vs. 1.70; *F*_1,36_ = 4.41; *p* = 0.043). There were no significant (*p* > 0.05) human contact treatment or housing system effects on the intensity of escape behaviour during tail clipping. There was a significant effect of human contact treatment on the duration of vocalisations at processing, with C piglets vocalising for longer in comparison to +HC piglets (6.30 vs. 4.47 s; *F*_1,36_ = 6.32; *p* = 0.018). There was also a tendency for C piglets to vocalise more often than +HC piglets (9.53 vs. 7.20; *F*_1,36_ = 3.62; *p* = 0.067). There were no significant (*p* > 0.05) housing system effects on vocalisations during processing, and no significant housing system × human contact interactions on any measurements at processing.

During administration of a vaccination at 3 wk of age, +HC piglets showed a lower intensity of escape behaviour in comparison to C piglets (mean score; 1.49 vs. 1.85; *F*_1,36_ = 8.96; *p* = 0.005; Table 1). However, there was no significant effect (*p* > 0.05) of housing system as seen during processing earlier in life. There was no significant (*p* > 0.05) housing system × human contact interaction effect on behavioural responses to vaccination at 3 wk of age.

#### 3.1.2. Physiological Responses to Processing

There were no significant (*p* > 0.05) effects of human contact treatment, housing system or the interaction between the two on plasma cortisol concentrations 45 min after processing at 3 d of age (Table 1).

### 3.2. Responses to Novelty and Humans in a Standard Test at 2 wk of Age

#### 3.2.1. Behavioural Responses to Capture before and after Testing

In comparison to C piglets, piglets from +HC litters showed a lower intensity of escape behaviour to capture from the home pen for testing (means score; 1.21 vs. 2.14; *F*_1,36_ = 15.2; *p* < 0.001) and to capture from the arena after testing had been conducted (1.48 vs. 2.13; *F*_1,36_ = 16.56; *p* < 0.001; Table 2). There were no significant (*p* > 0.05) housing system effects or housing system × human contact interaction effects on piglet responses to capture before or after the test.

#### 3.2.2. Behavioural Responses to Novel and Human Stimuli during the Test

There were no significant (*p* > 0.05) effects of human contact or housing system on the number of entries piglets made into different sections of the empty arena during the first minute of the test, although there was a tendency for FC piglets to enter more sections than LP piglets (5.30 vs. 4.16; *F*_1,36_ = 3.44; *p* = 0.072; Table 2). In comparison to LP piglets, FC piglets were faster to approach within 0.6 m of the traffic cone (15.5 vs. 30.8 s; *F*_1,36_ = 8.90; *p* = 0.005) and the human hand (20.9 vs. 34.9 s; *F*_1,36_ = 7.69; *p* = 0.009). Piglets from FC were also faster to physically interact with the traffic cone (22.8 vs. 34.5 s; *F*_1,36_ = 5.31; *p* = 0.027) and the human hand (31.0 vs. 42.9 s; *F*_1,36_ = 7.97; *p* = 0.008). There was also a tendency for FC piglets to be faster than LP piglets in approaching (24.1 vs. 34.8 s; *F*_1,36_ = 3.86; *p* = 0.058) and interacting (31.0 vs. 40.6 s; *F*_1,36_ = 3.30; *p* = 0.078) with the standing human. In comparison to C piglets, +HC piglets were faster to approach within 0.6 m of (24.0 vs. 34.9 s; *F*_1,36_ = 4.21; *p* = 0.048), and physically interact with (30.4 vs. 41.2 s; *F*_1,36_ = 4.49; *p* = 0.042), the standing human. However, there were no significant (*p* > 0.05) effects of human contact on responses to the traffic cone or human hand. There were also no significant (*p* > 0.05) effects of human contact or housing system on the time spent near or the number of tactile interactions with any stimuli, and no significant (*p* > 0.05) housing system × human contact interactions on any variables measured in the test.

### 3.3. Responses to Weaning

#### 3.3.1. Piglet Behaviour after Weaning

There were significant effects of housing system on several behaviours recorded from 15 min to 26 h after weaning. Higher proportions of pigs from LP than FC were observed to be upright and stationary (78.3 vs. 59.1%; *F*_1,31_ = 11.53; *p* = 0.002), upright and walking (40.3 vs. 32.0%; *F*_1,29.7_ = 5.63; *p* = 0.024), vocalising (2.93 vs. 1.93 %; *F*_1,28.7_ = 9.83; *p* = 0.004) and nosing a pen mate (22.2 vs. 17.8%; *F*_1,27.7_ = 4.93; *p* = 0.035; Table 3). There were also more pigs from LP nosing the pen floor at 24 h, 25 h and 26 h after weaning (13.2 vs. 6.75%; *F*_1,30_ = 6.32; *p* = 0.038). Housing system had no significant (*p* > 0.05) effects on the number of pigs engaging in play or aggressive behaviour. No tail biting behaviour was observed. There were no significant (*p* > 0.05) human contact effects or housing system × human contact interaction effects on pig behaviour after weaning.

#### 3.3.2. Physiological Responses after Weaning

There were no significant (*p* > 0.05) effects of human contact or housing system on plasma cortisol concentrations or on the ratio of neutrophil to lymphocyte cells 1.5 h after pigs were weaned (Table 4). However, 25 h post-weaning, there was a significant human contact × housing system interaction effect on plasma cortisol concentrations (*F*_1,28.2_ = 7.38; *p* = 0.012). Plasma cortisol concentrations were higher in C/LP pigs (44.3 ng/mL (33.9, 57.8)) compared to +HC/LP pigs (28.2 ng/mL (21.1, 37.6)) and C/FC pigs (28.6 ng/mL (21.7, 37.7)), but not +HC/FC pigs (37.8 ng/mL (29.7, 48.3)). Pigs from +HC litters also had higher plasma haptoglobin concentrations than C pigs 25 h after weaning (1130 vs. 697 µg/mL; *F*_1,28.3_ = 11.10; *p* = 0.002).

### 3.4. Sow Reproductive Performance and Piglet Growth, Injuries and Survival

#### 3.4.1. Injury Scores

LP piglets had higher injury scores than FC piglets at 2 wk of age during the lactation period (mean score; 1.30 vs. 0.850; *F*_1,36_ = 9.26; *p* = 0.004; Table 5), but there was no significant (*p* > 0.05) effect of housing system on injury scores obtained 2 d after weaning and mixing with unfamiliar pigs. Human contact had no significant (*p* > 0.05) effect on injuries at 2 wk of age during the lactation phase, however, after weaning and mixing with unfamiliar pigs +HC pigs had fewer injuries than C pigs (0.944 vs. 1.59; *F*_1,30_ = 10.68; *p* = 0.003). There were no significant (*p* > 0.05) housing system × human contact interaction effects on injury scores.

#### 3.4.2. Piglet Weights

There were no significant (*p* > 0.05) effects of human contact, housing system or the interaction between the two on piglet weights at 3 d or 18 d of age (Table 5).

#### 3.4.3. Piglet Survival during Lactation

There were no significant (*p* > 0.05) effects of housing system on the number of piglets born alive or the number of piglets stillborn (Table 5). The total number of piglets weaned was higher in FC than LP (9.50 vs. 8.25; *F*_1,36_ = 4.79; *p* = 0.035). There was no significant (*p* > 0.05) human contact effect or human contact × housing system interaction effect on the number of piglets weaned.

## 4. Discussion

This experiment studied the effects of farrowing and lactation housing systems and positive human interaction on stress resilience in piglets. Contrary to the expected findings, piglets reared in loose farrowing and lactation pens were slower to approach and interact with novel and human stimuli and were more reactive during capture by a stockperson early in life compared to piglets from farrowing crates. Piglets from loose pens also had higher injury scores during the lactation period and were more frequently observed to be active, vocalising, nosing the pen floor and nosing another pig after weaning. In both housing systems, providing regular opportunities for positive human interaction reduced piglets’ vocalisations and escape behaviour during husbandry procedures, and reduced the latency of piglets to approach and interact with a standing unfamiliar human.

Compared to piglets from loose pens, piglets from farrowing crates showed a lower intensity of escape behaviour during capture and iron administration by a stockperson at 3 d of age. While there were no effects of housing system on piglets’ behavioural responses to other husbandry procedures or to capture during behaviour testing, piglets from farrowing crates were faster to approach and initiate physical interaction with a novel object and an unfamiliar person at 2 wk of age, suggesting reduced fear of novelty and humans in piglets from farrowing crates compared to loose pens. Piglets from loose pens had increased opportunity for interaction with the sow, greater space and more complexity in their physical environment through pen features such as varied flooring and sloped walls. However, piglets from farrowing crates had substantially more visual, auditory and olfactory contact with stockpeople and adjacent pigs for several reasons. Firstly, the high walls of the loose pens restricted piglets contact outside the pen in general, to the extent that people were only visible to piglets when standing directly in front of the pen. Contact with neighbouring litters was also restricted until 2–3 wk of age when piglets were large enough to reach the pen windows, whereas piglets from farrowing crates were observed interacting over the wall with piglets from adjacent litters earlier in life. Secondly, there was more human traffic in the farrowing crate room as stockpeople required use of the aisles to access neighbouring sheds. Lastly, there were twice as many litters housed in the farrowing crate room, which increased the time stockpeople spent in this area conducting routine inspections and feeding. The design of the farrowing crate and PigSAFE Pen are likely to restrict contact between piglets and sows and contact between piglets and stockpeople conducting routine management, respectively. Further research therefore is required to test whether these restrictions contribute to fear responses of piglets to novelty and humans.

Piglets from farrowing crates may have been less fearful of humans due to increased opportunity for habituation to human presence. However, habituation is a stimulus-specific process [37], and responses to the novel object suggest piglets from farrowing crates were less fearful in general. Behaviour and physiological stress responses are affected by previous experiences coping with stress [38]. While exposure to severe stress early in life is damaging, repeated exposure to mild stress can assist animals in producing adaptive coping strategies for future stressful situations [2,3,4]. It may be that piglets in farrowing crates were provided with more opportunities to overcome mild stress through frequent and close visual exposure to stockpeople and their equipment and increased interaction with non-littermates. In contrast, piglets from loose pens were raised in a more isolated environment which may have contributed to increased fear of novelty and humans. Furthermore, sows in farrowing crates show fewer interactions with their piglets and reduced responsiveness to piglet vocalisations compared to sows in loose systems [7,8,9], and these reduced maternal responses may have resulted in the farrowing crate piglets having to be more reliant on learning to deal independently with stress. Whether these results are specific to the PigSAFE pens studied here or reflect piglet behaviour in other loose systems remains unknown. For example, differences in pen layout, room design and management may facilitate more contact with stockpeople and adjacent litters than in the PigSAFE pens in this experiment. While the effects of farrowing and lactation housing systems on piglets’ fear responses have not been widely examined, research by Chaloupkova et al. [12] reported similar findings to the present experiment where piglets from farrowing crates were more likely to make physical contact with an unfamiliar human compared to piglets from loose pens enriched with straw. Clearly further research on PigSAFE pens, as well as examination of other loose systems, is required to identify whether they similarly affect the fear responses of piglets to novelty and humans.

During processing at 3 d of age, piglets from the positive handling treatment vocalised for shorter durations and showed a lower intensity of escape behaviour during capture, iron injection and administration of an oral vaccination treatment. Positive human contact had no effect on the behavioural responses of piglets to tail clipping, although tail clipping was the last procedure to take place at processing and likely the most stressful. In a study by Muns and colleagues [23], piglets were gently touched on the head and snout during at least six suckling bouts on the first day of life. Each litter received at least 36 min of this treatment prior to processing, and Muns and colleagues reported that the treatment reduced escape behaviour in piglets during tail docking. In the present experiment, positive human contact litters had been exposed to the handling treatment for 9 min total prior to processing which may not have been enough to affect responses to painful events such as tail clipping. Nevertheless, both studies demonstrate that relatively small amounts of positive human interaction can improve the stress resilience of piglets to husbandry procedures imposed by stockpeople.

There were no human contact treatment effects on the cortisol response of piglets to processing at 3 d of age. However, effects on behavioural responses at processing were maintained, with positive human contact piglets showing less escape behaviour than routine contact piglets at 2 wk of age during capture for behaviour testing, and at 3 wk of age during capture and intramuscular vaccination. Furthermore, positive human contact piglets at 2 wk of age were faster to approach and physically interact with an unfamiliar human standing stationary. In line with several other studies, these results show that brief bouts of patting and stroking reduce fear of humans in pigs [20,21,26,39]. While positive human contact reduced piglets’ fear of the standing human, there were no effects on piglets’ behavioural responses to the same human extending their hand inside the test arena, possibly because the standing human stimulus more closely resembled the imposition of the positive human contact treatment. Additionally, piglets may not have associated the presentation of a hand inside the arena with a human. Aligning with these results, Muns et al. [23] found that positive human contact tended to reduce escape behaviour during capture at 15 d of age but had no effect on whether piglets interacted with a human hand. In the present experiment there were also no effects of human contact on responses to the novel arena or novel object, suggesting that the positive handling treatment reduced fear of humans but did not reduce general fearfulness. Earlier research on pigs [40] and poultry [41] showed similar effects.

Previous positive or negative interactions with humans have been shown to affect how pigs cope with weaning [19]. Pigs from routine human contact pens had more injuries after weaning which may indicate increased aggression or escape attempts from the pen, however, there were no effects of the positive handling treatment on pig behaviours post-weaning. There were however several effects of the farrowing and lactation housing system, with pigs from loose pens more frequently observed to be active, vocalising, nosing a pen mate and nosing the pen floor. This may have been due to pigs from loose pens experiencing a change in flooring after weaning. Pigs from farrowing crates were familiar with the slatted steel flooring in the post-weaning pens as it resembled the flooring in the farrowing crate system, while pigs from loose pens had only been exposed to plastic flooring prior to weaning. It is also possible that the reduced contact with stockpeople and non-littermates in the loose pen environment contributed to impaired stress resilience at weaning. Oostindger et al. [14] suggested that through increased opportunity for interaction with the sow, loose systems assist piglets in adapting to the post-weaning environment. The authors found that piglets from loose pens performed less belly nosing and manipulative behaviour and more play behaviour and food exploration after weaning compared to piglets from farrowing crates. These behaviours were sampled for 12 days post-weaning, whereas we only observed pigs until 26 h after weaning, which is a limitation of our study.

The physiological stress response to weaning in pigs can involve elevations of cortisol (plasma: 2 h post-weaning [42,43]; serum: 1 d post-weaning [44]) and an increase in the ratio of neutrophil to lymphocyte cells (2 h post-weaning [42], 1 d after weaning [45] and 3 d post-weaning [46]). In this experiment, there were no effects of housing system or human contact treatment on plasma cortisol concentrations or the ratio of neutrophil to lymphocyte cells 1.5 h post-weaning. However, at 25 h post-weaning, pigs reared in loose pens with routine human contact had higher plasma cortisol concentrations than pigs from +HC/LP and C/FC groups. One interpretation of this increased cortisol response at weaning in pigs from the loose pens is less stress resilience, although this effect was only found in pigs from loose pens with routine human contact. The cortisol concentrations of pigs from farrowing crates with positive human contact were intermediate and did not differ from the other treatment combinations.

In addition to the effects on plasma cortisol post-weaning, plasma haptoglobin concentrations 25 h after weaning were higher in pigs from the positive human contact treatment than from the routine handling treatment. Haptoglobin is one of several acute phase proteins (APP) released in response to infection, inflammation and stress [47], and has been shown to increase in pigs after weaning [48,49,50]. While the process behind the APP response to stress is not completely known, it is suggested that activation of the hypothalamic–adrenal axis and the release of glucocorticoids contributes to the synthesis of APP [18]. It was therefore surprising to find elevated cortisol concentrations in C/LP pigs but elevated haptoglobin concentrations in +HC pigs after weaning, although correlations between cortisol and haptoglobin are not always strong [51]. Haptoglobin also increases as part of the APP response to inflammation, and thus concentrations may increase due to injuries obtained from fighting with pen mates. However, positive human contact pigs had fewer injuries than routine human contact pigs 2 d after weaning, which suggests the elevated haptoglobin levels of positive human contact pigs were not a result of an inflammatory response. The study of the APP response to stress is somewhat new in pig welfare assessment and as such the effects of positive human contact on haptoglobin are not completely understood. More frequent sampling, as well as baseline measurements obtained prior to weaning, would provide further insight into the effects of housing systems and human contact on the physiological stress response of pigs to weaning.

There were no effects of housing system on the number of injuries pigs had 2 d after weaning. Results from previous studies assessing injuries at similar timepoints after weaning are varied, with pigs from farrowing crates reported to have more (4 d post-weaning [52]), less (3 d post-weaning [6]) or similar (6 h post-weaning [53]) injuries compared to pigs from loose pens. While injuries 2 d after weaning were similar between the housing systems in the present experiment, there were more injuries in loose pens than farrowing crates at 2 wk of age during the lactation period. Injuries are commonly sustained from fighting between pen mates [54], although they do not always correlate with aggressive behaviour [35] and may also be caused by collisions with the sow or pen fittings; both of which are more likely to occur in loose pens due to free movement of the sow and increased structural complexity of the pen. There were no effects of housing system or human contact treatment on piglet weights, the number of piglets born alive or the number stillborn, however, there were more piglets weaned from farrowing crates than loose pens. Previous experiments at the research site have shown that the number of pigs weaned is comparable between PigSAFE pens and farrowing crates [55,56]. However, sows in these experiments were of older parity and at least 2 kg of straw was provided in PigSAFE pens prior to farrowing. Piglet survival is likely to be higher in litters from more experienced sows and with the provision of bedding.

Only litters from primiparous sows were studied in the present experiment since there is evidence that piglet mortality is affected by changing farrowing system across parities [57]. Sows are generally more stressed at their first parturition than at subsequent ones [58,59], and stressors such as housing may also affect the sow which in turn may affect the piglets’ responses to stressors. However, the limited scientific literature on primiparous sows indicates little or no differences in stress, on the basis of cortisol concentrations, when housed in farrowing crates versus in loose systems (reviewed in [60]). The authors are presently studying piglets reared in farrowing and lactation housing systems with which their dams are familiar (i.e., systems they have previously farrowed in).

## 5. Conclusions

While sow and piglet welfare are generally considered to be superior in loose farrowing and lactation systems [6,9,14,61], the results from this experiment showed that piglets reared in loose pens displayed a higher intensity of escape behaviour when being captured during processing, and were slower to approach and physically interact with a novel object and an unfamiliar human compared to piglets from farrowing crates. Furthermore, piglets reared in loose pens had more injuries during lactation and were more likely to be upright, vocalising, nosing a pen mate and nosing the floor after weaning. While there were no effects of housing system on stress physiology 45 min after processing or 1.5 h after weaning, these results raise questions that require further research on the ability of piglets reared in loose pens to cope with stressors such as exposure to humans, novelty, husbandry procedures and weaning. Whether these effects are specific to the PigSAFE system studied here or are reflective of other loose systems is unknown. The PigSAFE system offers greater space and structural complexity and increased opportunities for interaction with the sow, however, in this experiment the PigSAFE environment was more restrictive in terms of piglets’ contact with humans and non-littermates. For piglets in farrowing crates, the increased visual, auditory and olfactory contact with stockpeople and other pigs may be adaptive and contribute to improved stress resilience by allowing piglets more opportunities to overcome mild stress. This experiment also provides evidence that regular positive human interaction reduces pigs’ fear of humans and husbandry procedures imposed by stockpeople. However, more research is clearly necessary to understand the effects of positive human contact on the injuries and stress physiology of pigs after weaning. The effects on stress physiology in particular highlight the need for a multifaceted approach to the welfare assessment of pigs. Additionally, whether the effects of the positive human contact treatment and those of the early housing system are sustained long-term requires further investigation.

## Figures and Tables

**Table 1:** Effects of human contact (+HC, positive human contact; C, routine human contact) and lactation housing system (FC, farrowing crate; LP, loose pen) on piglet responses to routine husbandry procedures at 3 d and 3 wk of age. Data represent means (95% confidence intervals). The number of vocalisations during processing and plasma cortisol concentrations 45 min after processing were logarithmically transformed prior to analysis; back-transformed data are presented.

Measurement	Human Contact		Housing System		*p*-Value		
	+HC	C	FC	LP	Human Contact	Housing System	Interaction
*Processing at 3 d of age*							
Escape behaviour score at capture and iron injection	1.32 (1.06, 1.57)	1.70 (1.44, 1.95)	1.32 (1.06, 1.57)	1.70 (1.44, 1.95)	**0.042**	**0.043**	0.691
Escape behaviour score at oral treatment administration	1.35 (1.16, 1.55)	1.68 (1.49, 1.88)	1.45 (1.25, 1.64)	1.59 (1.39, 1.79)	**0.026**	0.337	0.251
Escape behaviour score at tail clipping	1.61 (1.38, 1.84)	1.61 (1.37, 1.84)	1.69 (1.46, 1.91)	1.53 (1.29, 1.76)	0.970	0.340	0.126
Duration of vocalisations (s)	4.47 (3.41, 5.52)	6.30 (5.31, 7.29)	5.68 (4.62, 6.74)	5.08 (4.09, 6.07)	**0.018**	0.407	0.693
Number of vocalisations	7.20 (5.87, 8.84)	9.53 (7.88, 11.5)	7.94 (6.47, 9.75)	8.70 (7.19, 10.5)	0.067	0.577	0.618
Cortisol (ng/mL)	48.8 (40.1, 59.2)	51.2 (42.3, 61.8)	45.6 (37.7, 55.1)	54.6 (44.9, 66.3)	0.711	0.214	0.368
*Vaccination at 3 wk of age*							
Escape behaviour score at capture and vaccination	1.49 (1.32, 1.65)	1.86 (1.69, 2.03)	1.77 (1.60, 1.93)	1.57 (1.39, 1.74)	**0.005**	0.114	0.107

**Table 2:** Effects of human contact (+HC, positive human contact; C, routine human contact) and lactation housing system (FC, farrowing crate; LP, loose pen) on the behavioural responses of piglets to novelty and humans at 2 wk of age. Data represent means (95% confidence intervals).

Measurement	Human Contact		Housing System		Human Contact		
	+HC	C	FC	LP	Human Contact	Housing System	Interaction
*Response to capture*							
Escape behaviour score at capture from home pen pre-test	1.21 (0.881, 1.54)	2.14 (1.81, 2.47)	1.50 (1.17, 1.83)	1.85 (1.52, 2.18)	**<0.001**	0.148	0.911
Escape behaviour score at capture from arena post-test	1.48 (1.28, 1.68)	2.13 (1.93, 2.32)	1.91 (1.71, 2.11)	1.69 (1.49, 1.89)	**<0.001**	0.290	0.172
*Response to empty arena*							
Number of sections entered	4.53 (3.66, 5.39)	4.94 (4.08, 5.80)	5.30 (4.44, 6.16)	4.16 (3.30, 5.03)	0.517	0.072	0.763
*Response to traffic cone*							
Latency to approach 0.6 m (s)	20.5 (13.2, 27.8)	25.8 (18.7, 32.9)	15.5 (8.17, 22.8)	30.8 (23.8, 38.0)	0.291	**0.005**	0.107
Latency to interact (s)	27.3 (20.1, 34.6)	30.0 (23.0. 37.1)	22.8 (15.6, 30.1)	34.5 (27.5, 41.6)	0.559	**0.027**	0.105
Number of interactions	2.76 (2.02, 3.78)	2.67 (1.95, 3.66)	3.14 (2.29, 4.30)	2.35 (1.71, 3.22)	0.970	0.202	0.206
Time spent within 0.6 m (s)	20.6 (14.5, 26.7)	20.7 (14.7, 26.7)	22.2 (16.1, 28.3)	19.1 (13.1, 25.1)	0.982	0.448	0.218
*Response to human hand*							
Latency to approach 0.6 m (s)	26.8 (19.7, 33.9)	29.0 (22.1, 35.9)	20.9 (13.9, 28.0)	34.9 (28.0, 41.8)	0.643	**0.009**	0.534
Latency to interact (s)	34.5 (27.6, 41.2)	39.5 (32.7, 46.2)	31.0 (24.2, 37.8)	42.9 (36.0, 49.8)	0.231	**0.008**	0.799
Number of interactions	1.34 (0.813, 2.21)	1.23 (0.747, 2.03)	1.59 (0.964, 2.64)	1.04 (0.627, 1.71)	0.769	0.132	0.169
Time spent within 0.6 m (s)	14.2 (9.10, 19.4)	13.5 (8.55, 18.5)	15.8 (10.6, 20.9)	12.0 (7.02, 17.0)	0.863	0.326	0.445
*Response to standing human*							
Latency to approach 0.6 m (s)	24.0 (16.6, 31.4)	34.9 (27.7. 42.0)	24.1 (16.7, 31.4)	34.8 (27.6, 42.0)	**0.048**	0.058	0.420
Latency to interact (s)	30.4 (22.1, 38.7)	41.2 (32.9, 49.4)	31.0 (22.7, 39.3)	40.6 (32.2, 49.0)	**0.042**	0.078	0.546
Number of interactions	2.50 (1.39, 4.49)	1.79 (0.995, 3.21)	2.38 (1.32, 4.30)	1.87 (1.04, 3.38)	0.322	0.444	0.501
Time spent within 0.6 m (s)	20.7 (13.2, 28.1)	16.1 (8.68, 23.6)	20.4 (12.9, 27.8)	16.4 (8.85, 24.0)	0.355	0.423	0.393

**Table 3:** Effects of human contact (+HC, positive human contact; C, routine human contact) and lactation housing system (FC, farrowing crate; LP, loose pen) on pig behaviours post-weaning. Data represent the mean percent of pigs engaging in each behaviour at 15 min, 1 h, 2 h, 3 h, 4 h, 24 h, 25 h and 26 h post-weaning. The odds ratio (95% confidence interval) represents the likelihood of a +HC pig partaking in the behaviour over a C pig, or alternatively the likelihood of an LP pig partaking in the behaviour over an FC pig. When the odds ratio is 1, the chance of pigs from different treatments partaking in the behaviour is equal.

Measurement	Human Contact			Housing System			*p*-Value		
	+HC	C	Odds Ratio	FC	LP	Odds Ratio	Human Contact	Housing System	Interaction
Upright, stationary	71.9	67.1	1.25 (0.74, 2.12)	59.1	78.3	2.49 (1.47, 4.22)	0.639	**0.002**	0.365
Upright, walking	34.8	37.3	0.90 (0.67, 1.21)	32.0	40.3	1.43 (1.06, 1.93)	0.300	**0.024**	0.290
Vocalising	2.45	2.31	1.06 (0.81, 1.39)	1.93	2.93	1.53 (1.17, 2.00)	0.947	**0.004**	0.389
Nosing pen mate	19.4	20.4	1.06 (0.83, 1.36)	17.8	22.2	1.32 (1.03, 1.69)	0.461	**0.035**	0.420
Nosing pen floor ^1^	8.55	11.4	0.83 (0.43, 1.61)	6.75	13.2	2.03 (1.04, 3.96)	0.580	**0.038**	0.145
Play behaviour	0.621	1.04	0.59 (0.34, 1.04)	0.885	0.729	0.82 (0.47, 1.44)	0.124	0.649	0.299
Aggressive behaviour	1.52	1.24	1.24 (0.54, 2.82)	1.28	1.47	1.15 (0.50, 2.63)	0.684	0.666	0.115
Tail biting	0	0	-	0	0	-	-	-	-

^1^ Nosing the pen floor was rarely observed; only data from 24 h, 25 h and 26 h after weaning were included in the analysis.

**Table 4:** Effects of human contact (+HC, positive human contact; C, routine human contact) and lactation housing system (FC, farrowing crate; LP, loose pen) on the physiology of pigs after weaning. Data represent means (95% confidence intervals). All measurements were logarithmically transformed prior to analysis; back transformed data are presented.

Measurement	Human Contact		Housing System		*p*-Value		
	+HC	C	FC	LP	Human Contact	Housing System	Interaction
*1.5 h post-weaning*							
Cortisol (ng/mL)	32.1 (27.0, 38.1)	31.3 (26.8, 36.7)	30.1 (25.7, 35.2)	33.3 (28.1, 39.6)	0.946	0.393	0.988
Neutrophil to lymphocyte ratio	1.15 (0.966, 1.37)	1.12 (0.937, 1.348)	1.11 (0.934, 1.32)	1.16 (0.968, 1.40)	0.803	0.563	0.388
*25 h post-weaning*							
Cortisol (ng/mL)	33.7 (27.3, 41.4)	36.2 (29.4, 44.6)	34.0 (27.7, 41.6)	35.9 (28.9, 44.6)	0.652	0.597	**0.012**
Haptoglobin (µg/mL)	1130 (937, 1370)	697 (574, 846)	817 (679, 983)	966 (769, 1173)	**0.002**	0.212	0.139

**Table 5:** Effects of human contact (+HC, positive human contact; C, routine human contact) and lactation housing system (FC, farrowing crate; LP, loose pen) on piglet injury scores, weights and survival. Data represent means (95% confidence intervals).

Measurement	Human Contact		Housing System		*p*-Value		
	+HC	C	FC	LP	Human Contact	Housing System	Interaction
*Injuries*							
2 wk of age	1.03 (0.819, 1.23)	1.13 (0.919, 1.33)	0.850 (0.644, 1.06)	1.30 (1.09, 1.51)	0.503	**0.004**	0.185
2 d post-weaning	0.944 (0.675, 1.21)	1.59 (1.32, 1.85)	1.31 (1.05, 1.57)	1.22 (0.939, 1.50)	**0.003**	0.641	0.706
*Weights*							
3 d of age (kg)	1.55 (1.41, 1.69)	1.69 (1.55, 1.83)	1.60 (1.46, 1.74)	1.64 (1.50, 1.78)	0.162	0.677	0.895
18 d of age (kg)	4.43 (4.07, 4.78)	4.44 (4.08, 4.80)	4.45 (4.09, 4.81)	4.42 (4.06, 4.77)	0.953	0.896	0.827
*Piglet survival*							
Number born alive ^1^	-	-	11.8 (10.8, 12.8)	10.9 (9.88, 11.9)	-	0.226	-
Number stillborn ^1^	-	-	0.750 (0.260, 1.24)	1.05 (0.376, 1.72)	-	0.536	-
Number weaned ^2^	8.85 (8.06, 9.64)	8.90 (8.11, 9.69)	9.50 (8.71, 10.3)	8.25 (7.46, 9.04)	0.931	**0.035**	0.146

^1^ Only effects of housing system were tested as the positive handling treatment began at 1 d of age. ^2^ Includes fostered piglets.

## Data Availability

The data presented in this study are available on request from the corresponding author.
